# Clinical features and treatment outcomes of liver involvement in paediatric Langerhans cell histiocytosis

**DOI:** 10.1186/s12887-024-04764-5

**Published:** 2024-05-07

**Authors:** Xinshun Ge, Wenxin Ou, Ang Wei, Hongyun Lian, Honghao Ma, Lei Cui, Dong Wang, Liping Zhang, Xiaoman Wang, Lejian He, Rui Zhang, Tianyou Wang

**Affiliations:** 1 Hematology Center, Beijing Key Laboratory of Pediatric Hematology Oncology; National Key Discipline of Pediatrics (Capital Medical University); Key Laboratory of Major Disease in Children, Ministry of Education; Department of Hematology, Beijing Children’s Hospital, Capital Medical University, National Center for Children’s Health, Nanlishi Road No. 56, Xicheng District, Beijing, 100045 P.R. China; 2 Hematologic Disease Laboratory, Beijing Pediatric Research Institute; Hematology Center, Beijing Key Laboratory of Pediatric Hematology Oncology; National Key Discipline of Pediatrics (Capital Medical University); Key Laboratory of Major Disease in Children, Ministry of Education; Beijing Children’s Hospital, Capital Medical University, National Center for Children’s Health, Beijing, 100045 P. R. China; 3 Department of Ultrasonography, Beijing Children’s Hospital, Capital Medical University, National Center for Children’s Health, Nanlishi Road No. 56, Xicheng District, Beijing, 100045 P.R. China; 4 Department of Pathology, Beijing Children’s Hospital, Capital Medical University, National Center for Children’s Health, Nanlishi Road No. 56, Xicheng District, Beijing, 100045 P.R. China

**Keywords:** Children, Langerhans cell histiocytosis, Liver

## Abstract

Langerhans cell histiocytosis (LCH) is the most common histiocytic disorder in children, and liver involvement in LCH is rare. This retrospective study reported the clinical features and prognosis of patients with hepatic LCH. Liver involvement was defined by histopathological findings, liver dysfunction or abnormalities, or ultrasound imaging. A total of 130 patients (14.5%) with hepatic LCH out of 899 in the LCH population were enrolled. Patients with liver involvement had greater frequencies of skin, lung, hearing system, and haematologic system involvement, and hemophagocytic lymphohistiocytosis (*P*<0.001, 0.001, 0.002, 0.009, and <0.001, respectively). Overall survival and progression-free survival were lower in LCH patients with liver involvement than in those without liver involvement (*P*<0.001 and <0.001). In patients with liver involvement, the overall survival (OS) and progression-free survival (PFS) rates were lower in patients with cholangitis than in those without cholangitis (*P*<0.020 and 0.030). For the treatment response, the response rate of hepatic LCH patients to initial first-line therapy (*n*=89) was 22.5%. However, there was no significant difference in the response rate or recurrence rate between patients who shifted from first-line treatment to second-line treatment (*n*=29) or to targeted therapy (*n*=13) (*P*=0.453 and 1.000). The response rate of hepatic LCH patients who received initial second-line therapy (*n*=13) was 38.5%. Two of these patients subsequently experienced bone recurrence. The response rate of hepatic LCH patients who received initial targeted therapy (*n*=16) was 75.0%. Three patients subsequently experienced recurrence, including 2 in the bone and 1 in the liver and skin. A total of 39.3% of patients who received second-line treatment had severe myelosuppression (grade III-IV), and 50.8% had varying degrees of gastrointestinal events, whereas there was no severe toxicity in patients who received first-line treatment and targeted therapy. Four patients underwent liver transplantation because of liver cirrhosis. The patients’ liver disease improved within a follow-up period of 18-79 months. This study demonstrated that LCH with liver involvement, especially cholangitis, indicates a poor prognosis. Targeted therapy provides a good treatment response and less toxicity. However, it may relapse after withdrawal. Liver transplantation is still a reliable salvage option for patients with end-stage liver disease.

**Supplementary Information:**

The online version contains supplementary material available at 10.1186/s12887-024-04764-5.

## Background

Langerhans cell histiocytosis (LCH) is the most common histiocytic disorder and is characterized by the clonal expansion of pathological dendritic cells [1]. LCH mainly affects populations between 1 and 3 years of age [2]. The clinical course is heterogeneous, ranging from self-limited and indolent disease to rapid progression of multiorgan involvement. LCH has been redefined as being a tumour derived from haematopoietic myeloid progenitor cells. Liver involvement is observed in 10.1% to 19.8% of patients with LCH and is characterized by sclerosing cholangitis (SSC), which manifests as progressive destruction of the biliary tree by histiocytes [3]. LCH accompanied by haemophagocytic lymphoproliferative syndrome (HLH) may indirectly affect the liver. In such cases, a generalized activation of cellular immunity may lead to Kupffer cell hypertrophy and hyperplasia, with resultant hepatomegaly and elevated liver enzymes (but without direct infiltration). These indirect effects are entirely reversible with treatment [4]. The diagnosis of hepatic LCH is often difficult and delayed because of the absence of localized LCH. LCH patients with liver involvement could have a poor prognosis due to the high risk of the absence of timely and effective therapy, refractory disease, reactivation, and complications related to liver cirrhosis and portal hypertension [5]. The liver is considered a “risk organ” (RO) in LCH. For patients with liver involvement, the risk of death is three times greater than that for patients without liver involvement [6].

There have been few reports of hepatic LCH because it is a rare disorder, and its clinical characteristics and efficacy have yet to be fully investigated. Therefore, we conducted a large, single-centre, retrospective cohort study to evaluate the clinical characteristics and analyse the prognosis of paediatric patients with LCH and liver involvement, with an aim of reducing misdiagnosis and providing a treatment strategy.

## Materials and methods

### Patients

This retrospective study enrolled patients with LCH treated at Beijing Children's Hospital Affiliated with Capital Medical University between January 2016 and December 2022, and data, including demographic information, clinical features, laboratory examination, imaging results, treatment, and prognosis, were collected.

### Diagnostic and grouping criteria

#### LCH

The diagnosis of LCH was confirmed via pathology and immunohistochemistry positive for CD1a and/or CD207 (langerin) [7].

Liver involvement was defined as known LCH with one or more of the following: (1) histopathological findings of active Langerhans cell infiltration; (2) liver dysfunction, which was defined either by abnormal serum biochemical tests, such as hyperbilirubinemia, hypoproteinaemia (<55 g/L), hypoalbuminaemia (<25 g/L), elevated transaminase, and high γ glutamyl transpeptidase (GGT)/alkaline phosphatase (ALP), or by abnormal clinical manifestations, including ascites and oedema; (3) hepatomegaly (confirmed by ultrasound), which was defined as a liver edge >3 cm below the coastal margin at the midclavicular line [8]. According to the presence of liver involvement, the patients were divided into a liver group and a nonliver group.

SSC was defined as fulfilling all of the following criteria: (1) abnormal liver dysfunction, including two or more characteristics, such as increased direct bilirubin (DBIL), ALP, or γ-GGT; and (2) abdominal ultrasonography demonstrating bile duct wall thickening, irregularity, tortuosity, periportal abnormal echogenicity, bile duct dilatation or narrowing [9, 10]. According to the presence of SSC, patients in the liver group were further divided into a cholangitis group and a noncholangitis group.

LCH can be divided into single-system (SS) LCH and multiple-system (MS) LCH, according to the number of involved organs. According to RO involvement, the patients were divided into RO+ LCH and RO- LCH groups.

#### HLH

The diagnosis was in accordance with the criteria (HLH-2004) of the International Histiocyte Society [11].

### Treatments

LCH patients were treated with the systemic chemotherapy regimen BCH-LCH 2014 (http://www.chictr.org.cn, identifier: ChiCTR2000030457) based on the LCH-III and LCH-S-2005 treatments [12]. Vindesine is used because vinblastine is not available in China.

1. First-line therapy included the following: initial induction therapy involving vindesine (3 mg/m^2^/day) and prednisone (40 mg/m^2^/day); treatment for one or two weeks according to the treatment response; and maintenance therapy involving vindesine (3 mg/m^2^/day, every 3 weeks), prednisone (40 mg/m^2^/day, Day 1-5, every 3 weeks), and 6-mercaptopurine (50 mg/m^2^/day) (MS/RO+ LCH or CNS involvement). The total duration of the first-line therapy was 12 months, except for those non-CNS-risk multifocal skeletal LCH patients with good responses to the first initial therapy (6 months).
2. Second-line therapy (refractory to first-line therapy or relapse): four courses of intensive treatment involving one 5-day course consisting of cytarabine (150 mg/m^2^/day) at Days 1-5, cladribine (9 mg/m^2^/day) at Days 2-4, vindesine (3 mg/m^2^/day) at Day 1, and dexamethasone (6 mg/m^2^/day) at Days 1-5. Some patients did not receive cladribine due to economic factors; moreover, maintenance therapy included vindesine, prednisone and 6-mercaptopurine. The total duration of second-line therapy was 12 months.
3. For targeted therapy (http://www.chictr.org.cn, identifier: ChiCTR2000032844), patients who were refractory to chemotherapy or who relapsed and harboured the *BRAF V600E* mutation were treated with the BRAF inhibitor dabrafenib (2 mg/kg Q12H) for 1-1.5 years after providing informed consent.

### Gene mutation detection

LCH-related gene mutations in DNA from tissue and serum were determined by using the digital PCR method, as previously described [13]. The limit of detection of the assay was determined to be 0.1%.

### Treatment evaluation

Treatment response was evaluated according to the International LCH Study Group Criteria [7, 14]. Nonactive disease (NAD), active disease-better (AD-B), and AD-intermedia (AD-I) were defined as complete resolution, continuous regression of disease, or unchanged disease, respectively. AD-worse (AD-W) is characterized by the progression of signs or symptoms and/or the appearance of new lesions. Patients who responded to therapy were those who had NAD or AD-B. Recurrence was defined as the reappearance of signs and symptoms of AD after either complete disease resolution or after a period of disease control that persisted for >3 months on maintenance therapy. Refractorywas defined as the appearance of AD-W or AD-I during first-line treatment.

### Statistical analysis

IBM SPSS (26.0) software was used for the statistical analysis. Nonnormally distributed measurement data are expressed as the M (range), and normally distributed data are expressed as the mean ± SD. The t test or Mann‒Whitney test was used to determine differences between normally or nonnormally distributed measurement data. The chi-square test was used to determine differences between qualitative data. Univariate and multivariate logistic regression models were established to predict risk factors related to liver involvement in LCH patients. Survival rates were estimated with the Kaplan–Meier method, and subgroups were compared with the log-rank test. Cox regression was used to evaluate the effect of risk factors on survival. The overall response rate (ORR) was defined as the percentage of patients with NAD and AD-B among all of the patients. Progression-free survival (PFS) was estimated from the date of diagnosis to the date of one of the following events: AD-I or AD-W; relapse or reactivation after drug withdrawal or death (whichever event occurred first); and patients without events who were censored at the date of the last contact. Overall survival (OS) was defined as the time from the diagnostic date to the last follow-up. A *P* value<0.05 was considered to indicate statistical significance.

### Ethical statement

The study was conducted in accordance with the Declaration of Helsinki. This study was approved and was exempted from informed patient consent by the Ethics Committee of Beijing Children's Hospital, Capital Medical University.

## Results

### Clinical characteristics of patients with hepatic LCH

A total of 899 patients with LCH (the male-to-female ratio was 1.54:1) were enrolled, including 530 with SS LCH, 154 with MS RO+LCH, and 215 with MS RO-LCH. In the MS RO+LCH group, 130 patients had liver involvement, including 44 in the cholangitis group and 86 in the noncholangitis group. The liver is considered a risk organ; therefore, all patients with liver involvement were classified as having MS LCH. When we compared clinical features between patients with MS LCH with (*n*=130) or without (*n*=239) liver involvement, we found that patients with hepatic LCH were typically younger (*P* < 0.001). Furthermore, patients with liver involvement were more likely to develop fever (33.1%), rash (24.6%), splenomegaly (22.3%), and abnormal ear discharge (6.9%) (Table 1). Notably, patients with liver involvement had higher frequencies of skin, lung, hearing system, haematologic system involvement, and HLH (*P*<0.001, 0.001, 0.002, 0.009, and < 0.001, respectively) (Table 2). However, there were no patients with isolated liver involvement.

**Table 1:** Clinical features of LCH

	MS LCH with liver involvement (*n* = 130)	MS LCH without liver involvement (*n* = 239)	*P*
Gender (Male)	71	145	0.259
Age	2.25 ± 2.85	4.15 ± 3.79	**<0.001**
Fever	43	26	**<0.001**
Rash	32	29	**0.002**
Splenomegaly	29	6	**<0.001**
Ear discharge	9	6	**0.040**
Polyuria and polydipsia	7	13	0.982
Lymphadenopathy	6	17	0.343
White blood cells (×10^9^/L)	9.30 ± 4.78	8.85 ± 3.32	0.300
Neutrophils (×10^9^/L)	5.08 ± 4.01	4.32 ± 2.61	**0.030**
Hemoglobin (g/L)	98.5 ± 22.2	114.0 ± 17.22	**<0.001**
Platelets (×10^9^/L)	317.5 ± 200.0	365.0 ± 145.0	**0.010**
C-reactive protein (mg/L)	17 (1-183)	8 (1-204)	**<0.001**
Alkaline phosphatase (U/L)	265.0 (51.0-3398.0)	201.5 (49.0-487.0)	**<0.001**
Aspartate transaminase (U/L)	54.7 (6.5-1013.9)	30.4 (9.1-208.7)	**<0.001**
Alanine transaminase (U/L)	48.9 (4.7-623.9)	14.1 (4.0-89.2)	**<0.001**
γ-GGT (U/L)	109.3 (8.2-2663.3)	14.6 (6.0-113.4)	**<0.001**
Cholinesterase (U/L)	7891.0 (1307.0-14109.0)	7869.0 (1043.0-14080.0)	0.397
Total bile acid (umol/L)	9.70 (0.30-696.61)	3.18 (0.03-127.28)	**<0.001**
DBIL (umol/L)	1.41 (0.26-280.31)	0.98 (0.33-8.44)	**<0.001**
IFN-γ (pg/ml)	1.96 (0.00-190.59)	1.65 (0.00-182.12)	0.948
TNF-α (pg/ml)	2.32 (0.00-143.72)	1.78 (0.00-131.11)	0.199
IL-10 (pg/ml)	6.01 (0.41-77.62)	3.38 (0.00-40.76)	**<0.001**
IL-6 (pg/ml)	36.34 (1.19-2500.00)	11.64 (0.00-2182.8)	**<0.001**
IL-4 (pg/ml)	0.22 (0.00-5.47)	0.00 (0.00-8.97)	**0.032**
IL-2 (pg/ml)	0.00 (0.00-25.05)	0.43 (0.00-81.27)	**0.028**
CD4/CD8	1.75 (0.32-6.69)	1.62 (0.26-7.39)	0.452
Serum BRAF^V600E^ (%)	2.60 (0.11-26.62)	1.29 (0.12-15.25)	0.052
Tissue BRAF^V600E^ (%)	5.40 (1.00-37.00)	9.00 (0.74-36.00)	**0.020**

*γ-GGT* γ-glutamyl transferase, *DBIL* Direct bilirubin, *IFN* Interferon, *TNF* Tumor necrosis factor, *IL* Interleukin;

*P* values less than 0.05 were bold, which were considered statistically significant

**Table 2:** LCH with organ involvement

	MS LCH with liver involvement (*n* = 130)	MS LCH without liver involvement (*n* = 239)	*P*
Bone	104	209	0.057
Skin	91	101	**<0.001**
Lung	81	104	**0.001**
Audiometry system	43	45	**0.002**
Lymph node	37	74	0.617
Pituitary	34	72	0.421
Hematologic system	10	5	**0.009**
Diabetes insipidus	15	38	0.254
HLH	33	8	**<0.001**
Thyroid	22	28	0.163
Thymus	13	29	0.538
CNS (except pituitary)	10	24	0.456
Gastrointestinal tract	7	6	0.235

*HLH* Hemophagocytic lymphohistiocytosis;

*P* values less than 0.05 were bold, which were considered statistically significant

### Gene mutation test

In this study, 86 patients with liver involvement were detected with tissue gene mutations, and the tissue gene mutation rate was 90.7% (78/86, including 67 with *BRAF V600E* mutations and 11 with other mutations).The percentage of tissue with *BRAF V600E* mutations in the liver group was greater than that in the nonliver group (77.9% vs. 47.6%, *P*<0.001); however, the percentage of tissue with non-*V600E BRAF* mutations was lower (3.5% vs. 12.0%, *P*=0.025). Eighty-six patients with liver involvement were detected with plasma cell-free gene mutations, including 61 (70.9%) who were positive for the *BRAF V600E* gene mutation. Both the plasma cell-free *BRAF V600E* gene mutation rate and total gene mutation rate were greater in patients with liver involvement than in those without liver involvement (*P*<0.001) (Supplemental Table 1).

### Pathological result

All of the enrolled patients with hepatic LCH underwent histopathological examinations, and 9 of those patients underwent liver biopsies. A liver biopsy of typical hepatic LCH showed hepatocyte swelling and portal tract oedema with small bile duct proliferation and infiltration of massive neutrophils, lymphocytes, and a small number of eosinophils. Inflammatory cells infiltrate the small bile duct, thus leading to small bile duct sclerosis, partial "onion-like" changes, and infiltration by Langerhans cells with abundant cytoplasm and nuclear deviation in the local portal area. The utilized immunohistochemical stains were as follows: CD1a (+), Langerin (+), S-100 (+), CD68 (+), Ki-67 (10% +), CK7 (bile duct epithelium +), CD3 (lymphocyte +), and CD20 (lymphocyte +). The special utilized stains included D-PAS (+), Masson (+), and reticulated fibre (+) (Supplemental Figure 1).

### Imaging presentation of liver

All of the LCH patients with liver involvement had abnormal abdominal ultrasound findings at diagnosis. The most common abnormality on ultrasound was hepatomegaly (*n*=101, 77.7%), with the liver in the midline of the right clavicle located in the subcostal region of 2.47 (approximately 0.98-3.62) cm and a thickened spleen (*n*=91, 70.0%) with a thickness of 2.47 (approximately 0.98-7.70) cm. Moreover, there were several other imaging findings, such as abnormal echo (enhancement, inhomogeneity, and flaky hypoecho, among other findings) (*n*=74, 56.9%), splenomegaly (*n*=59, 45.4%), Glisson capsule thickening (*n*=48, 36.9%), bile duct involvement (bile duct wall thickening, dilatation or narrowing, and tortuosity) (*n*=44, 33.8%) and hilar lymph node enlargement (*n*=41, 31.5%) (Supplemental Figure 2).

### Treatment response

**Fig. 1:**
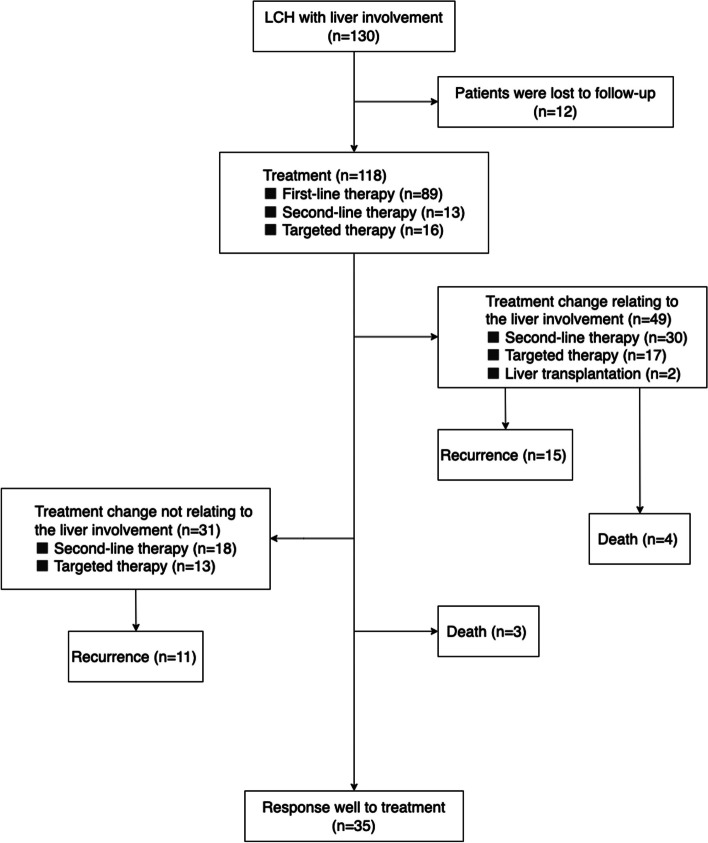
Flow diagram of treatment for LCH patients with liver involvement

The treatment of patients with liver involvement is shown in Fig. 1. Among the 89 patients who received initial first-line treatment, 20 (22.5%) had improved liver lesions. Sixty-nine (77.5%) patients had either progression, relapse, or no improvement of risk organs and were shifted to second-line or targeted treatment, 42 (60.9%) of whom had liver involvement. Twenty-nine patients were switched to second-line treatment due to liver involvement. Among these patients, 20 (69.0%) experienced improvement in the liver, 7 (24.1%) were shifted to targeted therapy due to liver progression (4 with improved liver involvement, 1 with liver cirrhosis, 1 with successful liver transplantation, and 1 who died of liver cirrhosis), and 2 (6.9%) died of HLH. Thirteen patients were switched to targeted therapy due to liver involvement. Among them, 11 (84.6%) had improved liver lesions, 1 died of liver cirrhosis, and 1 developed liver cirrhosis. There was no significant difference in the liver improvement rate or relapse rate between patients who were switched from first-line therapy to second-line therapy or to targeted therapy (the liver improvement rate: 69.0% vs. 84.6%, *P*=0.453; the relapse rate: 20.7% vs. 15.4%, *P*=1.000). There was also no significant difference in the 2-year OS rate between the two treatment groups (89.0% ± 6.0% vs. 66.7% ± 27.2%, *P*=0.862). Among the 13 patients who received initial second-line treatment, 5 (38.5%) had improved liver lesions, 2 (15.4%) had no significant change in liver involvement, 1 (7.7%) developed liver cirrhosis, 4 (30.8%) were shifted to targeted therapy due to liver progression, and 1 (7.7%) died of liver cirrhosis. During the follow-up, two patients (15.4%) treated with initial second-line therapy presented with bone relapse (the times from withdrawal to relapse were 7.5 months and 12.4 months, respectively). Among the 16 patients who received initial targeted therapy, 12 (75.0%) patients had improved liver lesions, 2 (12.5%) received liver transplantation due to liver progression, and 2 (12.5%) had no significant change in liver involvement. However, three patients (18.8%) who were treated with initial targeted therapy relapsed, with two patients relapsing in the bone region and 1 patient relapsing in the liver and skin regions (the times from withdrawal to relapse were 1.6 months, 3.0 months, and 1.0 months, respectively).

No severe adverse events were observed during first-line treatment or targeted therapy. Nevertheless, 39.3% of patients treated with second-line treatment had severe myelosuppression (grade III-IV), and 50.8% of patients had various grades of gastrointestinal events, including nausea, vomiting, and diarrhoea.

**Fig. 2:**
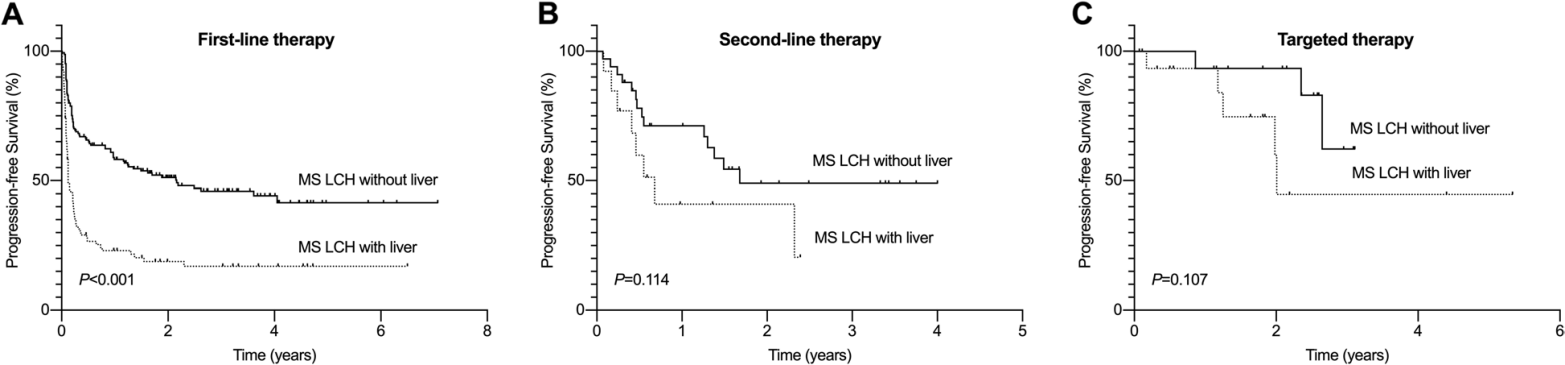
Comparison of PFS for LCH patients with or without liver involvement who underwent different initial therapies. **A** PFS for patients with multisystem LCH who received initial first-line therapy. **B** PFS for patients with multisystem LCH who received initial second-line therapy. **C** PFS for patients with multisystem LCH treated with initial targeted therapy. PFS: progression-free survival

We also compared the treatment responses of patients with MS LCH among different initial treatments. A total of 223 patients in the MS LCH without liver group were followed up, including 165 with first-line treatment, 34 with second-line therapy, 16 with targeted therapy, and 8 with symptomatic treatment. Among patients with MS LCH who received initial first-line treatment, those with liver involvement had a lower 2-year PFS rate than those without liver involvement (17.0% ±4.3% vs. 41.6%±4.9%, respectively; *P*<0.001). In MS LCH patients who received initial second-line therapy or targeted therapy, there was no significant difference in the 2-year PFS rate between patients with or without liver involvement (20.5% ±16.3% vs. 49.0% ±10.1%, *P*=0.114; 44.8%±18.1% vs. 62.2% ±19.9%, *P*=0.107) (Fig. 2).

### Prognostic analysis

Follow-up was continued until December 2022. Seventy-seven patients were lost to follow-up (12 in the liver group and 65 in the nonliver group), with a median follow-up of 2.55 (0.08-7.08) years. Seven patients in the liver group died, with 2 dying from secondary HLH and severe infection, 2 dying from respiratory failure caused by lung involvement, and 3 dying from portal hypertension caused by liver involvement. All of the patients in the nonliver group were alive.

**Fig. 3:**
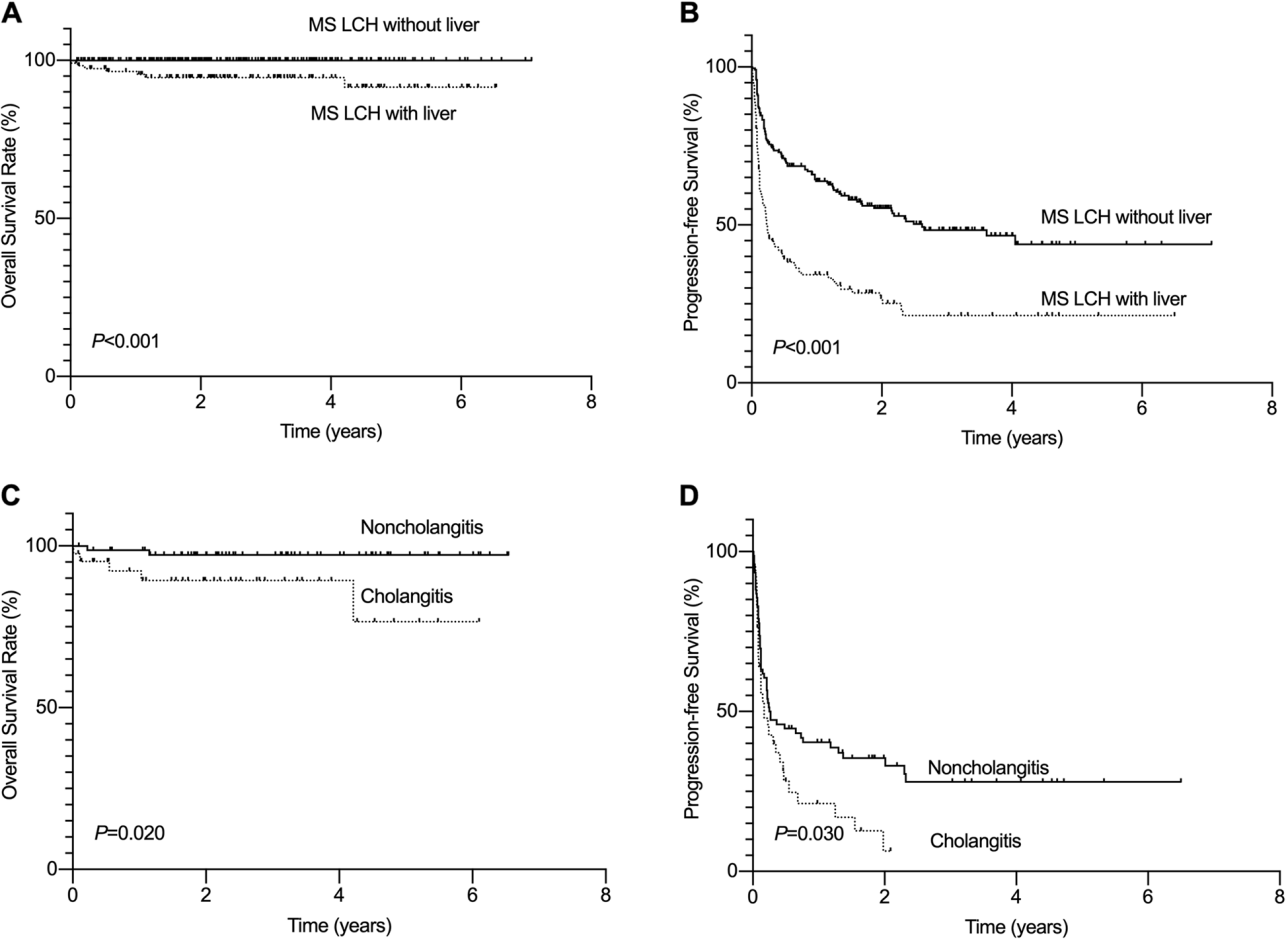
Comparison of survival rates for LCH patients. **A** OS of multisystem LCH patients with or without liver involvement. **B** PFS of multisystem LCH patients with or without liver involvement. **C** OS of patients with hepatic LCH with or without cholangitis. **D** PFS of patients with hepatic LCH with or without cholangitis

We explored the prognosis of patients with MS LCH. The 2-year OS rate in the MS LCH with liver group was significantly lower than that in the MS LCH without liver group (91.6% ±3.6% vs. 100%, respectively; *P*<0.001). Similarly, the 2-year PFS rate in the MS LCH with liver group was significantly lower than that in the MS LCH without liver group (21.3%±4.6% vs. 43.9%±4.7%, respectively; *P*<0.001). Cox regression analysis demonstrated that liver involvement significantly impacted the survival of patients with MS LCH (*P*<0.001, hazard ratio: 2.349, 95% CI: 1.755-3.144). Further analysis of subgroups in the MS LCH with liver group demonstrated that the 2-year OS and PFS rates of patients in the cholangitis group were significantly lower than those in the noncholangitis group (76.6% ±12.6% vs. 97.2% ±2.0%, *P*=0.020; 6.4% ±5.5% vs. 28.0% ±5.9%, *P*=0.030) (Fig. 3). Thirty-three patients (75.0%) in the cholangitis group experienced disease progression/recurrence. Among these patients, 22 (66.7%) had liver involvement. Fifty-one patients (59.3%) in the noncholangitis group had disease progression/recurrence, 29 (56.9%) of whom had liver involvement.

In addition, four patients in the MS LCH with liver group underwent liver transplantation because of liver cirrhosis, with a follow-up time of approximately 18-79 months. Their last evaluations were all AD-B. Two patients received reduced targeted therapy, and 2 only received oral antirejection drugs. Notably, one patient developed intrahepatic relapse at 18 months after liver transplantation and resolved LCH disease after second-line treatment in our centre, which included the use of cladribine and cytarabine. Currently, there has been no relapse after 46 months of withdrawal.

The evaluation of three patients who died of liver involvement in our cohort demonstrated that they all had high levels of ALP, γ-GGT, and DBIL (ALP >900 U/L, γ-GGT >500 U/L, and DBIL >40 µmol/L). Additionally, the multivariate analysis demonstrated that increased levels of ALT, γ-GGT, total bile acid, and DBIL were independent risk factors for liver progression or relapse (*P*<0.001, <0.001, =0.009 and 0.008, respectively) (Table 3).

**Table 3:** Univariate and multivariate analysis

	Univariate analysis			Multivariate analysis		
	*P* value	OR	95%CI	*P* value	OR	95%CI
ALP	<0.001	30.462	9.152~101.390	0.099	3.376	0.796~4.321
AST	<0.001	4.850	3.042~7.729	0.478	0.757	0.351~1.634
ALT	<0.001	10.143	5.925~17.362	**<0.001**	3.407	1.754~6.618
γ-GGT	<0.001	13.038	7.757~21.914	**<0.001**	5.221	2.844~9.587
Cholinesterase	0.924	0.920	0.166~5.093	-	-	-
Total bile acid	<0.001	9.486	5.418~16.608	**0.009**	2.603	1.264~5.361
TBIL	<0.001	12.360	4.171~36.625	0.156	0.461	0.268~1.798
DBIL	<0.001	68.949	9.263~513.246	**0.008**	21.280	2.214~204.537

*OR* Odds ratio, *CI* Confidence interval, *ALP* Alkaline phosphatase, *AST* Aspartate transaminase, *ALT* Alanine transaminase, *γ-GGT* γ-glutamyltransferase, *TBIL* Total bilirubin, *DBIL* Direct bilirubin

## Discussion

In our study, patients with liver involvement accounted for 14.5% of the LCH patients in the same time, which is similar to previous reports [3]. Liver involvement is exclusively observed in patients with MS LCH, and patients may present with hypoproteinaemia, oedema, hepatomegaly, and hyperbilirubinemia [15]. We found that MS LCH patients with liver involvement were more prone to developing multisystemal symptoms such as fever, rash, splenomegaly, and abnormal ear discharge than were those without liver involvement. In terms of the involved organs, patients with liver involvement had increased frequencies of skin, lung, and hearing system involvement, as well as HLH. Currently, LCH is generally redefined as being an inflammatory myeloid neoplasia driven by activating mutations in the MAPK pathway. A French LCH cohort demonstrated that 89.2% of patients with hepatic LCH carried a *BRAF V600E* mutation, which is related to activation of the MAPK pathway, increased resistance to first-line treatment and increased reactivation [14]. Our study also demonstrated that the *BRAF V600E* mutation rate in the tissue and plasma of patients with liver involvement was greater than 70%. In addition, we also identified several rare gene mutations, such as *non-V600E BRAF*, *MAP2K1*, and *ARAF*. The percentage of *non-V600E BRAF* mutation-positive tissues in the liver group was lower than that in the nonliver group (*P*=0.025). Previous research has reported that patients with *BRAF V600E* mutations are prone to having multiple systems and organs at risk [16].

Liver involvement is associated with poor prognosis in LCH patients, especially those with SSC [17]. The 2-year OS rate in the MS LCH with liver group was significantly lower than that in the MS LCH without liver group (*P*<0.001). In addition, the 2-year PFS rate in the MS LCH with liver group was only 21.3% ±4.6%. Further multivariate analysis demonstrated that increased levels of ALP, γ-GGT, total bile acid, and DBIL were independent risk factors for liver progression/relapse during treatment. Thus, clinicians need to closely monitor liver function during therapy.

Currently, standard therapy for LCH patients with liver involvement has yet to be established. Yi et al. [8] showed that 44.4% of LCH patients with liver involvement presented with different degrees of improvement in biochemistry and imaging studies after treatment with the conventional vinblastine-prednisone-etoposide combination; moreover, two patients died of multiple organ failure secondary to worsening liver dysfunction. Our study showed that in MS LCH patients who received initial first-line therapy, patients with liver involvement had a shorter PFS (*P*<0.001). The treatment of 60.9% of patients in the liver group changed due to liver events during first-line treatment, thus suggesting that for patients with hepatic LCH, conventional first-line treatment was only effective for some patients, and the liver lesions in nearly 2/3 of LCH patients did not respond well to first-line treatment. There was no significant difference in the liver improvement rate between patients who were switched from first-line to second-line therapy and those who were shifted to targeted therapy (*P*=0.453). Among patients who received initial second-line or targeted therapy, 38.5% and 75.0%, respectively, had improved liver lesions. The therapeutic response of patients with *BRAF* mutations to targeted therapy may not be poor. Patients who were RO+ had worsened responses to treatment, and the level of the *BRAF V600E* mutation was associated with the extent of LCH disease [18]. Therefore, this type of patient may be ideal for targeted therapy. However, 39.3% and 50.8% of the patients in our study experienced different degrees of severe myelosuppression and gastrointestinal events, respectively, after second-line treatment. Thus, targeted therapy appears to be safe for reducing toxicity (to some extent). Improvements in symptoms and liver function were rapidly obtained after the patients accepted the target medicine. Nevertheless, studies have shown that half of patients experience relapse or progression, which may be attributed to reactivation of the MAPK pathway or too short a duration of targeted treatment [19, 20]. Three of the patients who received initial targeted therapy in our study relapsed, and the time from withdrawal to recurrence was short (approximately 1-3 months). Thus, the optimal strategy for targeted therapy should be extensively explored. Notably, the effectiveness of second-line treatment and targeted therapy also showed that liver involvement was reversible, and timely diagnosis and treatment could reduce liver cirrhosis.

Previous studies and liver biopsies have demonstrated that Langerhans cells have remarkable selectivity for bile ducts [21]. Studies have reported a 25% response to chemotherapy in LCH patients with SSC [22]. We found that the 2-year OS and PFS rates in the cholangitis group were significantly lower than those in the noncholangitis group (*P*=0.002 and 0.030, respectively). In our study, 4 patients who developed liver cirrhosis and 3 who died of sclerosis presented with bile duct involvement at disease onset. Notably, bile duct destruction is irreversible. Despite the resolution of active Langerhans cell infiltration, biliary duct injury continues to progress [4]. Caruso et al. [23] reported of a child who was diagnosed with LCH via skin and lymph node biopsies. The patient first presented with hepatomegaly but not cholangitis. On follow-up during chemotherapy, the patient had gradually increased liver enzymes and developed SSC and biliary cirrhosis. Therefore, it is necessary to identify and diagnose LCH as soon as possible and to perform early treatment to avoid irreversible liver involvement. Nevertheless, there are few studies on treating LCH patients with liver involvement. The optimal therapy remains unclear, and treatment-related side effects and economic costs still need further assessment.

Due to the fact that SSC is usually progressive, liver transplantation is a reliable salvage therapy for LCH patients with end-stage liver disease [3]. Several groups have performed orthotopic liver transplantation in children with end-stage biliary diseases secondary to LCH-related SSC, and they reported an OS of 87% with a mean follow-up time of 3.4 years [24]. Five patients who received effective chemotherapy, as reported by Chen et al. [25], underwent liver transplantation. A follow-up time of 2 to 67 months demonstrated that liver function was stable for a long period of time without serious complications or liver reactivation. Four patients received liver transplantation in our study. Their final evaluations were AD-B, with a follow-up period of approximately 18-79 months. One patient experienced intrahepatic reactivation 18 months posttransplant and then received first-line and second-line treatment. Currently, there has been no relapse after 46 months of withdrawal. The disease activity of LCH patients with end-stage liver involvement must be closely monitored after liver transplantation. Moreover, chemotherapy is still safe and effective for patients with liver reactivation posttransplantation. However, the optimal timing of liver transplantation is a matter of debate, as performing liver transplantation too early may result in the recurrence of the primary disease. Moreover, delayed transplantation may hamper optimal chemotherapy for reactivation in diseased livers [17].

We performed the largest cohort study on LCH patients with liver involvement; however, the current study had several limitations. First, the data excluded dynamic monitoring in disease, such as the level of liver function, liver size, and the *BRAF V600E* gene mutation rate after treatment. Moreover, liver transplantation has yet to be performed in our centre. All of the transplantation recordings in our study were provided by patients, and there may be a lack of accurate assessments of hepatic LCH before and after transplantation.

## Conclusion

LCH patients with liver involvement consistently present with a high *BRAF V600E* mutation rate in tissue and plasma. Liver involvement, especially cholangitis, indicates a poor prognosis in patients with LCH. Conventional first-line therapy may not be effective in patients with hepatic LCH, and second-line therapy and targeted therapy could be considered treatment options for such patients. It is important to note that targeted therapy may provide a promising treatment option with less toxicity, but there is a possibility of recurrence after withdrawal. In the future, the therapeutic regimen for hepatic LCH requires further research.

### Supplementary Information

**Supplementary Material 1.** 

## Data Availability

The datasets used and analysed during the current study available from the corresponding author on reasonable request.

## Abbreviations

LCH: Langerhans cell histiocytosis
RO: Risk organs
SSC: sclerosing cholangitis
HLH: hemophagocytic lymphohistiocytosis
γ-GGT: γ-glutamyl transpeptidase
ALP: alkaline phosphatase
NAD: Nonactive disease
AD-B: active disease-better
AD-I: AD-intermedia
AD-W: AD-worse
ORR: Overall response rate
PFS: Progression-free survival
OS: Overall survival
