# Supplementary material for: Clinical features and treatment outcomes of liver involvement in paediatric Langerhans cell histiocytosis
Source: BMC Pediatr. 2024 May 7;24:316. doi: 10.1186/s12887-024-04764-5 (PMC11077857; doi:10.1186/s12887-024-04764-5)
Supplement: Supplementary file 1 — Supplementary Material 1. [file 12887_2024_4764_MOESM1_ESM.docx]

**Supplemental Table 1 Gene mutation analysis**

|  | MS LCH with  liver involvement (n=130) | MS LCH without  liver involvement (n=239) | *P* |
| --- | --- | --- | --- |
| Tissue gene mutation analysis | 86（66.2%） | 166（69.5%） | 0.515 |
| BRAF V600E mutation | 67（77.9%） | 79（47.6%） | **<0.001** |
| Non-V600E BRAF mutations | 3（3.5%） | 20（12.0%） | **0.025** |
| MAPK21 | 5（5.8%） | 22（13.3%） | 0.086 |
| Other mutations | 3^a^（3.5%） | 17^b^（10.2%） | 0.060 |
| Total mutations | 78（90.7%） | 138（83.1%） | 0.104 |
| Plasma gene mutation analysis | 86（66.2%） | 173（72.4%） | 0.211 |
| BRAF V600E mutation | 61（70.9%） | 64（37.0%） | **<0.001** |
| Non-V600E BRAF mutations | 0（0.0%） | 1（0.6%） | 1.000 |
| MAP2K1 | 1（1.2%） | 6（3.5%） | 0.430 |
| Total mutations | 62（72.1%） | 71（41.0%） | **<0.001** |

^a^: 1 MEK1, 1 ARAF, 1 TP53;

^b^: 4 ARAF, 1 ALK, 1 APC, 1 DDR1, 1 DICER1, 1 FGFR1, 1 GNA11, 1 IFNGR1, 1 KIT, 1 KRAS, 1 NRAS, 1 PDGFB, 1 ROS1, 1 TP53;

P values less than 0.05 were bold, which were considered statistically significant.


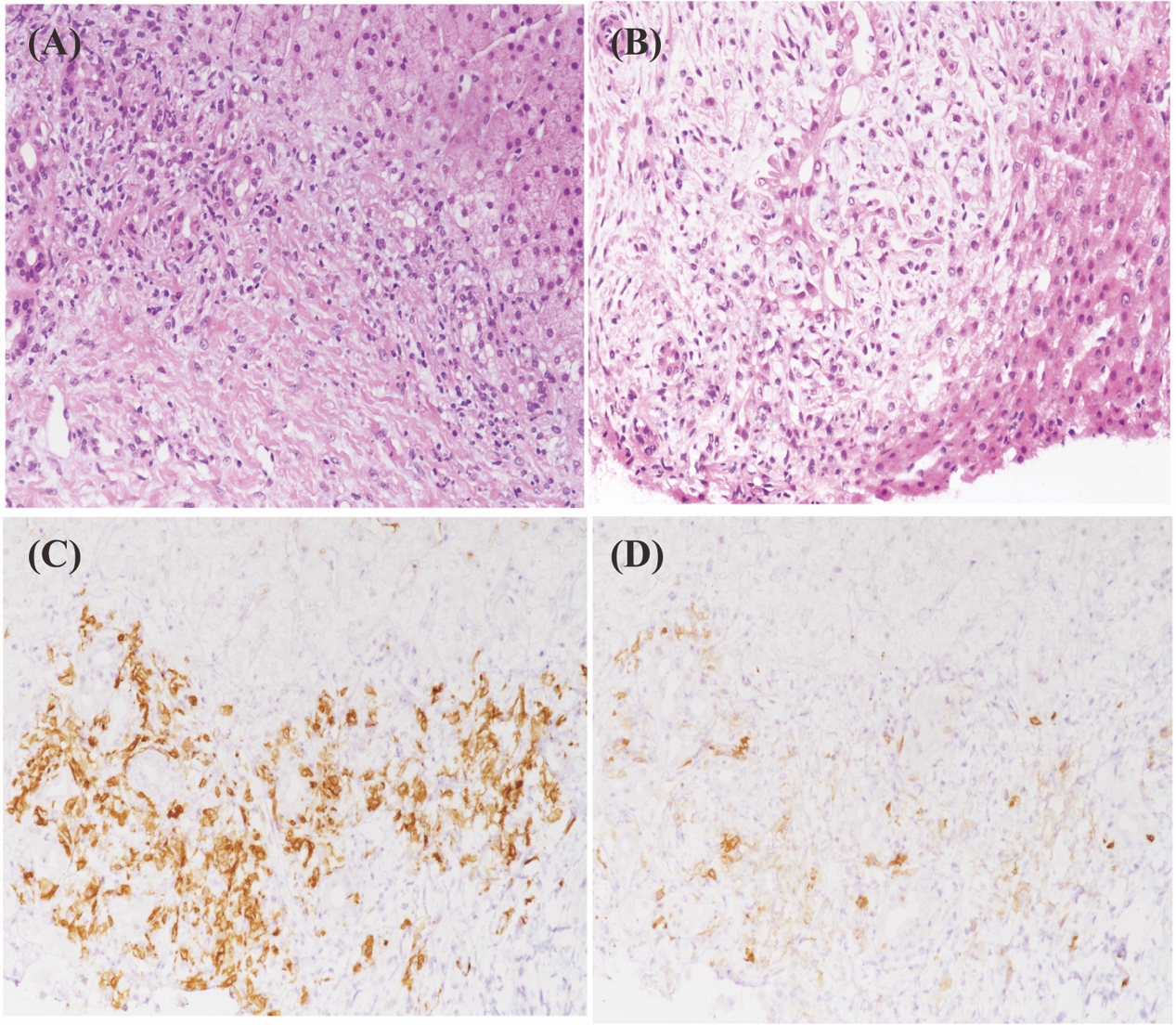


**SUPPLEMENTAL FIGURE 1** A biopsy of the liver in patients with hepatic LCH. (A-B) Hepatocyte swelling with the infiltration of massive neutrophils, lymphocytes, and a small number of eosinophils (hematoxilin and eosin, x100); (C) Langerin positive (x400); (D) CD1a positive (400x).


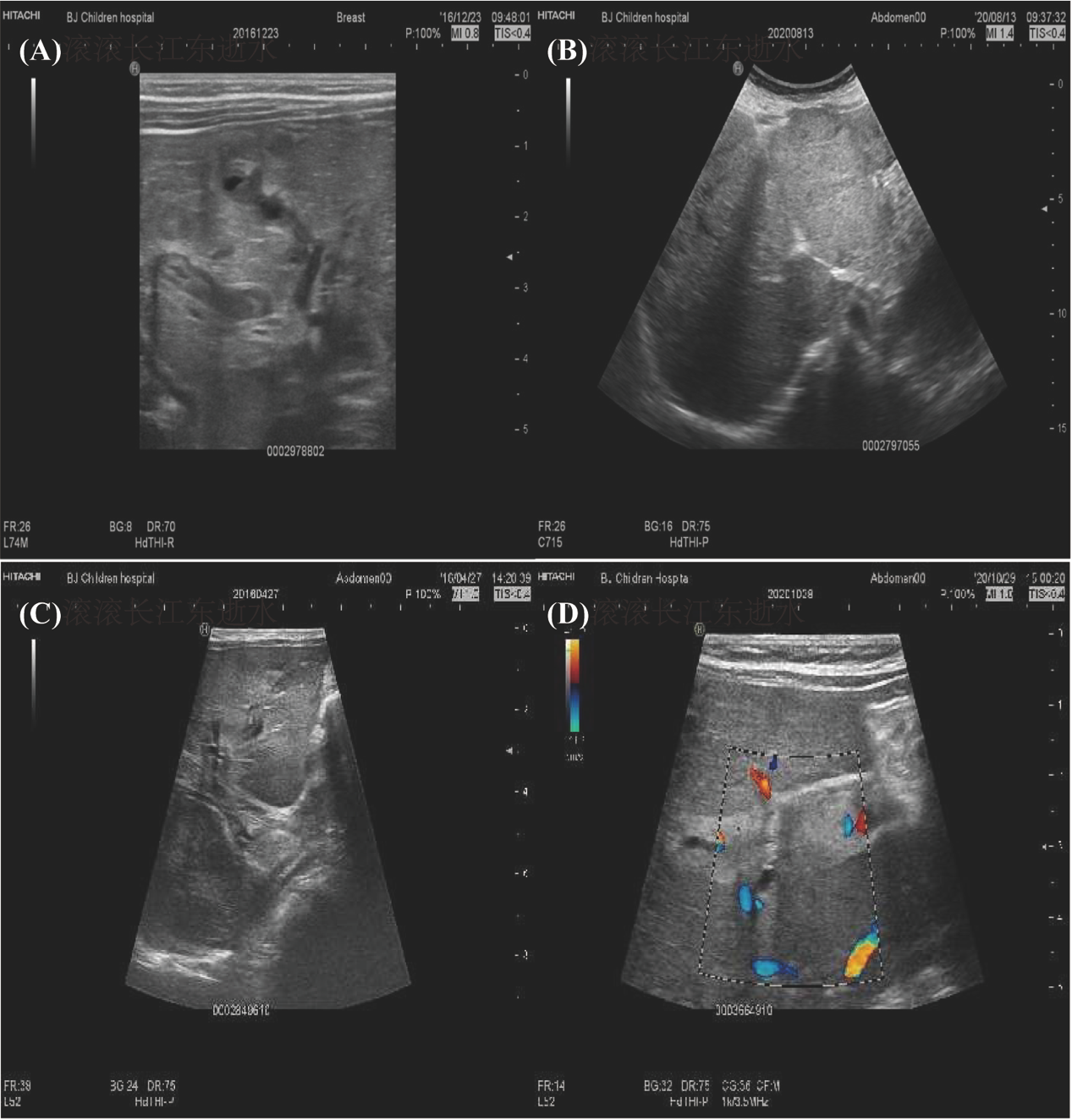


**SUPPLEMENTAL FIGURE 2** Ultrasonography of hepatic LCH. (A) The diameter of bile duct in hilar area was about 0.4cm; the diameter of common bile duct was about 0.8cm; there were scattered deposits in the enlarged bile duct; (B) Glisson capsule had uneven thickening, some of which were nodular, especially in the left lateral lobe, with a range of 1.0×0.9cm. (C-D) The intrahepatic bile ducts were widened; the width of the left hepatic duct was about 0.25cm; the width of the right hepatic duct was about 0.15cm; the intrahepatic and extrahepatic bile duct walls were diffusely thickened; the width of intrahepatic bile duct wall was about 0.4cm, and the width of common bile duct wall was about 0.27cm.
